# Subgaleal Hematoma in a Female With Normal Coagulation Tests

**DOI:** 10.1155/crh/5481806

**Published:** 2025-08-30

**Authors:** Sajjad Ataei-Azimi, Mettine H. A. Bos, Hossein Rahimi, Hassan Mansouritorghabeh

**Affiliations:** ^1^Hematology and Oncology Section, Department of Internal Medicine, Ghaem Hospital, Mashhad University of Medical Sciences, Mashhad, Iran; ^2^Department of Internal Medicine, Division of Thrombosis and Hemostasis, Einthoven Laboratory for Vascular and Regenerative Medicine, Leiden University Medical Center, Leiden, the Netherlands; ^3^Central Diagnostic Laboratories, Ghaem Hospital, Mashhad University of Medical Sciences, Mashhad, Iran; ^4^Noor Medical Laboratory, Jame Jam Building, 3rd Aref Avenue, Aref Street, Ahmad Abad Street, Mashhad, Iran

**Keywords:** clot solubility test, factor XIII activity, factor XIII deficiency, intracranial hemorrhage

## Abstract

Factor XIII (FXIII) deficiency is a rare coagulopathy with an estimated prevalence of approximately 1 in 1 to 2 million, affecting males and females with equal frequency. FXIII plays a critical role in hemostasis by stabilizing fibrin clots through covalent cross-linking of fibrin monomers, thereby conferring mechanical resistance and durability to the clot structure. Clinically, FXIII deficiency presents with a spectrum of hemorrhagic manifestations including bleeding from the umbilical cord, intracranial hemorrhage, recurrent miscarriages, menorrhagia, epistaxis, gingival bleeding, and poor wound healing. Despite significant bleeding symptoms, routine primary hemostasis screening tests are typically within normal limits since FXIII acts downstream of clot formation. The clot solubility in 5-molar urea is widely used, especially in resource-limited settings. An 11-year-old female patient presented with symptoms including vomiting, lethargy, severe headache, and a subgaleal hematoma. Neurosurgical intervention confirmed intracranial hemorrhage. Her medical history was notable for neonatal umbilical cord bleeding, hematomas, and postdental extraction bleeding. Despite these clinical features, multiple clot solubility tests yielded normal results. Subsequent quantitative assessment of FXIII by chromogenic assay performed on the CS-5100 system revealed a markedly decreased FXIII activity level of 12.4%. This discrepancy highlights the limited insensitivity of the clot solubility test in detecting FXIII deficiency. Therefore, accurate diagnosis of FXIII deficiency necessitates a combined diagnostic approach incorporating both clot solubility testing and specific quantitative FXIII activity measurement. The clot stability test, despite its limitations in detecting FXIII deficiency, is frequently employed in developing countries for screening reduced FXIII levels due to its simplicity. However, the current findings indicate that in patients suspected of FXIII deficiency, accurate diagnosis necessitates the performance of both a clot stability test (5 M urea test) and a specific FXIII activity assay. A comprehensive medical and family history is fundamental to the clinical and laboratory approach to patients presenting with bleeding tendencies. Notably, a subset of patients exhibiting bleeding symptoms may exhibit normal findings on initial first-line hemostasis screening assays.

## 1. Introduction

Patients presenting with bleeding symptoms, particularly those with a history of hemorrhagic episodes since early childhood and a positive family history of similar bleeding manifestations, are likely to have an inherited bleeding disorder. It is essential to obtain a detailed family history, which should include an assessment of consanguinity in the parents. Prothrombin time (PT) and activated partial thromboplastin time (APTT) are insufficient for the detection of factor XIII (FXIII) deficiency. Relying solely on these assays can lead to a failure in diagnosing FXIII deficiency (FXIIID). The initial case of FXIIID identified using the clot stability assay was reported by Duckert in 1960 [1, 2]. Patients with severe FXIIID may predispose with fatal bleeding. Many cases show bleeding from the umbilical cord in the neonate period. Despite the inherited FXIIID with symptoms from childhood, some patients with no history of bleeding may experience acquired FXIIID secondary to inhibitory antibodies [3]. In FXIII-deficient plasma, fibrin clots exhibit lysis upon exposure to 5 M urea or 1% monochloroacetic acid in the clot solubility test. While this assay possesses significant utility for identifying severe FXIIID cases, it demonstrates limited sensitivity. Specifically, the clot stability assay utilizing 5 M urea can only detect residual FXIII activity levels below 5%. Substituting 2% acetic acid improves the assay's sensitivity, enabling detection of FXIII activity below 10%.

## 2. Case Presentation

An 11-year-old female patient was admitted to a pediatric hospital on August 11, 2024, with vomiting, lethargy, decreased level of consciousness, and severe headache. Initial laboratory investigations revealed no notable abnormalities (Table 1). A cranial computed tomography (CT) scan identified the presence of a subgaleal hematoma in the left cerebral hemisphere. An intracranial hematoma occurred spontaneously in the absence of any antecedent head trauma. Then a spontaneous subgaleal hematoma was documented, measuring 15 × 40 mm in maximal cross-sectional diameters in the left parietal region. Emergency neurosurgical intervention was performed for evacuation of the intracranial hemorrhage. Following a 6-day hospitalization, she was discharged in stable condition. Throughout the perioperative period, fresh frozen plasma (FFP) was administered with a dosage of 20 mL/kg of body weight.

On postdischarge day 1, the patient returned with fever and seizures, requiring admission to the intensive care unit (ICU). Blood cultures confirmed bacteremia. Management included surgical drainage of an abscess and antibiotic therapy. Prior to tranexamic acid (10 mg/kg body weight) was administered via slow intravenous infusion.

A postdischarge blood sample was referred to a private accredited medical laboratory (Noor Laboratory) for investigation of the hemorrhagic diathesis etiology. The treating physician requested a comprehensive coagulation factor assessment; all measured factors were within the reference range (Table 1). The FXIII assay, conducted using the clot solubility method, was normal (> 60 min). Her pediatric hospital medical records documented two prior normal clot stability tests, performed in her home province and in Tehran.

When her father requested that we, as a private accredited medical laboratory, intervene in the diagnostic process for her coagulopathy. At this stage, a parental interview was conducted in the laboratory setting to acquire a detailed medical history. The patient was the firstborn child; subsequent siblings exhibited no history suggestive of bleeding tendency. The parents reported a consanguineous marriage. The maternal history indicated no miscarriage. Significant hemorrhagic events were recalled, including umbilical cord bleeding, a hematoma posttraumatic cranial trauma (secondary to swing impact), and excessive bleeding following a tooth extraction during infancy. Additionally, the mother reported a family history of FXIIID in her older sister, who experienced a fatal hemorrhage approximately 20 years prior.

Given the discordance between clinical hemorrhagic manifestations and initial normal laboratory findings, diagnostic sensitivity limitations were suspected. Therefore, subsequent FXIII activity assessment using the chromogenic method on a CS-5100 system (Sysmex, Kobe, Japan) revealed a markedly reduced activity level of 12.4%.

The patient was subsequently placed on a prophylactic therapy with plasma-derived FXIII concentrate (Fibrogammin P, CSL Behring, Germany), which successfully prevented further bleeding episodes.

The follow-up during the 12-month postdiagnosis period was associated with no bleeding episode. This clinical course aligns with the recognized phenotype FXIII, where FXIII activity levels ranging from 5% to 30% are commonly associated with variable bleeding severity [4].

## 3. Discussion

The Scientific and Standardization Committee (SSC) of the International Society on Thrombosis and Haemostasis (ISTH) recommends against utilizing the clot solubility assay due to the significant influence of multiple confounding variables on test results [5]. Nevertheless, compliance with this guideline remains variable among hemostasis laboratories, largely attributable to technical challenges, economic limitations, and the lack of standardized protocols across institutions. In the current case, a plasma FXIII activity level of 12.4% was detected in concomitance with a severe bleeding phenotype. This observation may reflect a consumption coagulopathy secondary to hemorrhagic events or the presence of a truncated FXIII variant that maintains antigenicity yet is functionally impaired in supporting effective hemostasis.

FXIII exhibits an extended half-life ranging from 11 to 14 days. Consequently, a prophylactic dosing regimen of 10 to 20 units/kg administered at 4- to 6-week intervals is recommended to prevent spontaneous hemorrhagic events. In pregnant individuals with FXIIID, initiation of prophylactic therapy before 5 to 6 weeks of gestation is advised to reduce miscarriage risk. Sustaining FXIII activity levels above 10% can be effectively achieved through weekly plasma infusions of 250 units until the 22nd week of gestation [6]. Owing to the heterogeneity of genetic mutations affecting FXIII structure and function, molecular genetic analysis constitutes an adjunct for definitive diagnosis.

Approximately 3%–5% of all inherited bleeding disorders are classified as rare bleeding disorders (RBDs). Among these, FXIIID has an estimated global prevalence of approximately 1 in 2 million individuals. A markedly higher prevalence of FXIIID has been reported in southeastern Iran, largely attributed to the high rate of consanguineous marriages in the region [7]. The disorder has also been documented in other Middle Eastern countries, including Pakistan and India [8], although southeastern Iran remains a recognized epidemiological hotspot for FXIIID. FXIII plays a pivotal role in the coagulation cascade by catalyzing the cross-linking and stabilizing of fibrin clots following fibrin monomer formation [7]. Due to its position in the coagulation pathway, patients with FXIIID typically exhibit normal PT and APTT results. The deficiency results in the formation of unstable fibrin clots that are more prone to proteolytic degradation by the fibrinolytic system. Clinically, FXIIID patients commonly present with bleeding manifestations, such as umbilical cord bleeding, menorrhagia, epistaxis, hemarthrosis, muscular and articular hemorrhages, gingival bleeding, and prolonged postoperative bleeding [8, 9]. Umbilical cord hemorrhage has been observed in approximately 80% of neonates diagnosed with FXIIID [6]. Intracranial hemorrhage and recurrent miscarriage, attributable to hemorrhagic complications, constitute the primary causes of morbidity and mortality in postnatal FXIIID individuals. Females of reproductive age with FXIIID may not display a generalized bleeding diathesis, frequently presenting exclusively with recurrent miscarriages. Delayed diagnosis and inadequate management of bleeding episodes may result in severe outcomes and consequences, ranging from life-threatening hemorrhages to milder chronic sequelae such as iron deficiency anemia.

The implementation of a chromogenic assay for the detection of FXIIID is critical, particularly in patients presenting with clinical presentations indicative of FXIIID. Exclusive reliance on the clot stability assay may fail to identify all cases of FXIIID and is associated with notable diagnostic limitations. Several studies suggest that the utilization of insufficiently sensitive diagnostic tests can lead to misdiagnosis of FXIIID, which may have fatal consequences. Furthermore, early diagnosis in patients exhibiting bleeding diathesis enables timely therapeutic intervention, thereby reducing hospital length of stay and associated healthcare costs. Prophylactic treatment in patients with FXIIID has been demonstrated to significantly decrease the incidence of bleeding events.

## Figures and Tables

**Table 1:** Laboratory findings in the case with factor XIII deficiency.

Laboratory test	Patient	Reference range
WBC	12.40 × 10^3^/mL	4–11 × 10^3^/mL
RBC	4.62 × 10^6^/Micl	4.2–5.4 × 10^3^/Micl
Hb	12.7 g/dL	12.1–15.7 g/dL
HCT	38.2%	36%–48%
MCV	90.2 fL	80–100 fL
MCH	29 pg/cell	27–31 pg/cell
MCHC	34 g/dL	33–36 g/dL
Platelet count	264 × 10^3^	150–450 × 10^3^/Micl
PMN	81.7%	40%–50%
Lymphocyte	15.9%	20%–40%
Monocyte	2.4%	< 10%
ESR	16	20 mm/h
PT	13.3 s	12–14 s
APTT	28.5 s	30–40 s
CRP	6.5 mg/L	< 10 mg/L
Total protein serum	8.61 g/dL	5.7–8 g/dL
Ferritin	88.2 ng/mL	13–150 ng/mL
VWF: Ag	102.7%	50%–150%
VWF: activity	127%	41%–125.9%
FXIII	Sufficient	< 60 min = deficient 50–120 min = borderline, > 120 min = sufficient
FXIII chromogenic method	12.4%	60%–146%
Protein C	132%	70%–140%
Fibrinogen	425 mg/dL	200–400 mg/dL
D-Dimer	571 ng/mL	< 500 ng/mL
Antithrombin III	118%	80%–120%
FVIII activity	138%	50%–150%
FIX activity	134%	50%–150%
FV activity	142%	50%–150%
FX activity	121%	50%–150%
FVII activity	97%	50%–150%
FXI activity	107%	50%–150%
FXII activity	145%	50%–150%

*Note:* Hb: hemoglobin, HCT: hematocrit, Ag: antigen, F: factor, mg/dL: milligram per deciliter, ng/mL: nanogram per milliliter, min: minute.

Abbreviations: RBC, red blood cell; VWF, von Willebrand factor; WBC, white blood cell.

## Data Availability

The data that support the findings of this study are available from the corresponding author by e-mail.
